# Pharmacological Neurorescue in a Paclitaxel-Induced Chemobrain Model

**DOI:** 10.3389/fnbeh.2021.736003

**Published:** 2021-09-21

**Authors:** Peng Chen, Fuchao Chen, Benhong Zhou

**Affiliations:** ^1^Department of Pharmacy, Renmin Hospital of Wuhan University, Wuhan, China; ^2^School of Pharmaceutical Sciences, Wuhan University, Wuhan, China; ^3^Sinopharm Dongfeng General Hospital, Hubei University of Medicine, Shiyan, China

**Keywords:** pharmacological, paclitaxel, chemobrain model, memory, mechanism

## Introduction

Cancer is one of the most common metabolic diseases in the clinical setting. More than 30% of the cancer patients receive chemotherapy, despite the misgivings of oncologists (Wang et al., 2021). Nevertheless, the majority of patients receiving chemotherapy, such as doxorubicin or paclitaxel, are at risk for neurodegeneration in the brain, leading to a condition known as chemobrain or chemo fog (Wahdan et al., 2020). In particular, chemobrain is a deteriorating condition that adversely affects the lives of cancer survivors. Its pathological features include memory loss, decreased speed in functional execution, and increased feelings of anxiety, as compared to patients without a history of cancer (Dan, 2019). Most of the studies indicate chemobrain as a multifactorial disease, resulting from a variety of mechanisms, such as oxidative stress, neuroinflammation, DNA damage, neuronal apoptosis, and aberrant hippocampal neurogenesis (Erken et al., 2020). However, the specific molecular mechanisms contributing to its pathology remain unclear. Furthermore, recent studies have found that the long-term lithium treatment in the mouse brain can produce neuroprotective and neurotrophic effects (Hermelink, 2019). Notably, the ameliorative effect of lithium on paclitaxel-induced cognitive impairment in mice has attracted much interest due to its anti-oxidative, anti-inflammatory, and anti-apoptotic actions. Although these studies have revealed causal relationships between lithium and chemobrain, its underlying mechanism has yet to be fully understood.

A new study by Nguyen et al. (2021) in molecular neurodegeneration reported that after paclitaxel exposure, behavioral tasks consistent with a persistent cognitive deficit, such as memory loss, increased feelings of anxiety, and impaired locomotor activity, were observed in the treated mice compared with control mice. Conversely, these behavioral deficits were rescued with lithium pretreatment, suggesting that lithium administration following paclitaxel exposure can exert a protective effect against chemobrain-associated memory impairment. Moreover, upon performing Golgi–Cox staining of the brain hemispheres and subsequent Sholl analysis of the dentate gyrus granule cells, the results were found to be similar to the findings in the previous behavioral experiments of the paclitaxel-induced, short-term, and memory impairment model. Furthermore, this study proved a valuable finding that the neuron complexity tended to be stable after an initial burst of cognitive disorder in the progression of chemobrain. Thus, lithium rescue of cognitive function in the paclitaxel-exposed model was mainly accomplished *via* hippocampal neuron complexity enhancement (Pease-Raissi et al., 2017).

**FIGURE 1:**
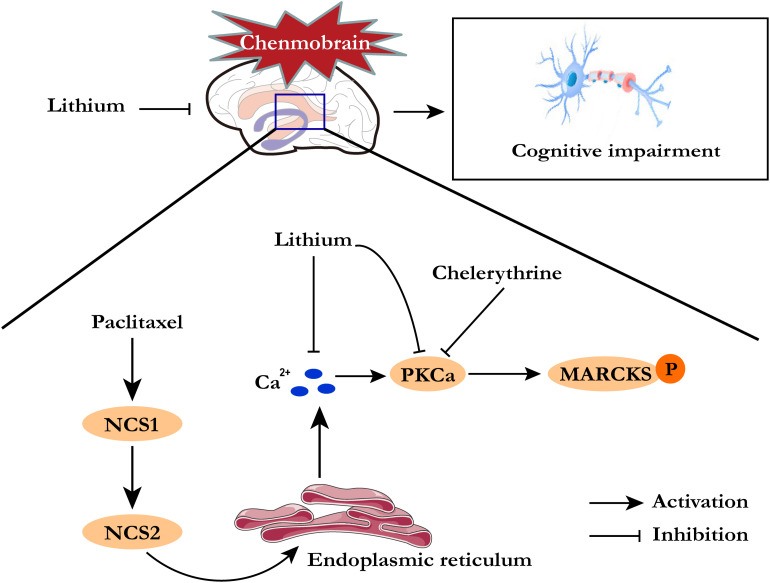
Lithium pretreatment for paclitaxel-induced cognitive deficits.

Using the western blotting techniques, the authors demonstrated PKCα upregulation, whereas other proteins involved in the inositol trisphosphate receptor (InsP3R) pathway, such as InsP3R1, NCS1, and phospholipase C (PLC-β1), remained unchanged between the paclitaxel-treated and control groups. The authors also reported an increased protein expression of PKCα at 24 h after paclitaxel injection in mice, with the phosphorylated form of the PKC substrate myristoylated alanine-rich C-kinase substrate showing an increasing trend as well. This provides evidence that PKCα may possibly be involved in the initiation of the chemobrain pathogenesis. However, as a crucial limitation to the study, the authors did not evaluate the paclitaxel-induced chemobrain mice between 24 h and 4 weeks, which was the time when PKCα activation was noted to have increased and reached a plateau (Olopadea et al., 2019). Furthermore, they additionally investigated the effects of chelerythrine pretreatment (a PKC inhibitor) and paclitaxel on hippocampal and neuronal morphology. Similar to the previous observations, chelerythrine treatment increased the complexity and dendritic length of hippocampal granule cells and the apical dendrites of cortical neurons, whereas the basal dendrites were spared. These results further emphasized that chelerythrine and lithium act in a similar pathway to rescue the paclitaxel-induced, short-term, and memory impairment (Figure 1). However, it should be noted that the corresponding mechanism research was not well-grounded in this study. To confirm whether PKCα was a key target for paclitaxel-induced neuronal injury, this gene should be knocked down or knocked out, and then, the neurological function and synaptic integrity of neurons in the chemobrain mice model should be investigated (Mcintosh et al., 2010). Another limitation to this study was that the researchers did not detect other cytokines that can mediate PKCα in the paclitaxel-induced chemobrain model, such as growth factors and transcription factors. Therefore, in the future studies, additional chemobrain-associated biomarkers at various time points should be evaluated to provide further evidence to illuminate the role of InsP3R calcium pathway responses in the pathophysiology of chemobrain.

Notably, the most interesting finding by Nguyen et al. (2021) was that these results provided newer insights for the non-surgical therapies targeting the InsP3R calcium pathway for the treatment of chemotherapy-induced cognitive impairment, especially in the delayed operative treatment. They asserted that prompt treatment for paclitaxel-induced cognitive impairment was very important, opening the discussion on whether drug intervention at earlier stages (e.g., at 24 h, when the increase in PKCα hyperactivity was most pronounced) could contribute to the normalization of chemobrain changes. Additionally, the substantial studies have provided evidence that hippocampal neurogenesis alterations were also associated with chemobrain pathogenesis (Park et al., 2018). These previous data suggested that other cell types, such as neural stem cells, could contribute to the pathophysiology of chemobrain. Therefore, a comprehensive targeting of multiple factors should be considered in the context of diseases in further studies, as this would be the most effective in the long-term restoration of paclitaxel-induced cognitive impairment.

In summary, this study was very interesting and gives further evidence that the InsP3R calcium pathway in paclitaxel-induced cognitive impairment cannot be neglected despite diagnostic uncertainty. Moreover, early paclitaxel exposure was found to mediate calcium response and PKCα upregulation and was noted to play an important role in the chemobrain pathogenesis. It was also found that the regulation of InsP3R-dependent calcium release in the early stages of chemobrain can help prevent or delay disease progression. Additionally, Nguyen et al. (2021) proposed that utilizing the animal models that better reflect and simulate chemobrain in humans are needed, such as aged or tumor-bearing mice receiving intravenous (IV) paclitaxel infusion mimicking the standard treatment regimens. Notably, it was found that no matter which way was chosen to establish the chemobrain model, alterations in the blood–brain barrier (BBB, a barrier between the brain and blood and it controls the substances transporting from blood to the barrier) were reported upon paclitaxel treatment *in vivo*, whereas *in vitro* assays revealed a temporary paclitaxel-associated barrier disruption (Kaiser et al., 2014). Therefore, a possible interpretation of chemotherapy-related cognitive decline could take into consideration the capacity of the drug to cross the BBB (Park et al., 2018). In this regard, researchers need to take into account that repeated paclitaxel administration in chemotherapy protocols could promote crossing of the barrier (Bernatz et al., 2019). For all the findings of the study, further studies using neuroimaging and molecular tools, such as single-cell whole-genome amplification and sequencing, can help expound the neuroimmune mechanism and cell types involved in chemobrain pathology. Furthermore, the findings of these studies, in addition to the present findings of this study, could eventually help develop better diagnostic and therapeutic intervention strategies for chemobrain.

## Author Contributions

PC, FC, and BZ conceptualized and designed the study. PC wrote the manuscript. PC and FC designed the figures. BZ reviewed the manuscript.

## Conflict of Interest

The authors declare that the research was conducted in the absence of any commercial or financial relationships that could be construed as a potential conflict of interest.

## Publisher’s Note

All claims expressed in this article are solely those of the authors and do not necessarily represent those of their affiliated organizations, or those of the publisher, the editors and the reviewers. Any product that may be evaluated in this article, or claim that may be made by its manufacturer, is not guaranteed or endorsed by the publisher.
